# Diversity and seasonal dynamics of bacterial community in indoor environment

**DOI:** 10.1186/1471-2180-8-56

**Published:** 2008-04-08

**Authors:** Helena Rintala, Miia Pitkäranta, Mika Toivola, Lars Paulin, Aino Nevalainen

**Affiliations:** 1 Environmental Health Department, National Public Health Institute, P.O. Box 95, 70701 Kuopio, Finland; 2 DNA Sequencing Laboratory, Institute of Biotechnology, P.O. Box 56, 00014 University of Helsinki, Finland

## Abstract

**Background:**

We spend most of our lives in indoor environments and are exposed to microbes present in these environments. Hence, knowledge about this exposure is important for understanding how it impacts on human health. However, the bacterial flora in indoor environments has been only fragmentarily explored and mostly using culture methods. The application of molecular methods previously utilised in other environments has resulted in a substantial increase in our awareness of microbial diversity.

**Results:**

The composition and dynamics of indoor dust bacterial flora were investigated in two buildings over a period of one year. Four samples were taken in each building, corresponding to the four seasons, and 16S rDNA libraries were constructed. A total of 893 clones were analysed and 283 distinct operational taxonomic units (OTUs) detected among them using 97% sequence similarity as the criterion. All libraries were dominated by Gram-positive sequences, with the most abundant phylum being *Firmicutes*. Four OTUs having high similarity to *Corynebacterium*-, *Propionibacterium*-, *Streptococcus*- and *Staphylococcus*- sequences were present in all samples. The most abundant of the Gram-negative OTUs were members of the family *Sphingomonadaceae*, followed by *Oxalobacteraceae*, *Comamonadaceae*, *Neisseriaceae *and *Rhizobiaceae*.

The relative abundance of alpha- and betaproteobacteria increased slightly towards summer at the expense of firmicutes. The proportion of firmicutes and gammaproteobacteria of the total diversity was highest in winter and that of actinobacteria, alpha- and betaproteobacteria in spring or summer, whereas the diversity of bacteroidetes peaked in fall. A statistical comparison of the libraries revealed that the bacterial flora of the two buildings differed during all seasons except spring, but differences between seasons within one building were not that clear, indicating that differences between the buildings were greater than the differences between seasons.

**Conclusion:**

This work demonstrated that the bacterial flora of indoor dust is complex and dominated by Gram-positive species. The dominant phylotypes most probably originated from users of the building. Seasonal variation was observed as proportional changes of the phyla and at the species level. The microflora of the two buildings investigated differed statistically and differences between the buildings were more pronounced than differences between seasons.

## Background

We spend most of our lives in different indoor environments; in homes, day-care facilities, schools and workplaces, and are continuously challenged by the microbial content of these environments. Traditionally, infections are considered as the main health effect of bacteria; however, these micro-organisms can affect our health in many other ways. During infancy, contact with bacteria in the environment leads to development of the indigenous human microflora, the importance of which for human health has been acknowledged but is still not fully understood [1]. Microbial exposure in childhood also prevents the development of allergic conditions [2,3]. Later in life, microbial exposure in indoor environments can also affect human health; for example, moisture damage in a building can lead to increased indoor microbial levels and this can have adverse health effects [4].

New questions concerning environmental microbial exposure have emerged lately. For example, the numbers of community acquired MRSA infections among healthy individuals have risen rapidly in recent years [5], showing that there are reservoirs of this pathogen outside of hospitals. Although infectious agents normally cannot persist for a long time in air, a recent study has shown that antibiotic-resistant *Staphylococcus aureus *is common in indoor air of normal residential buildings [6]. Against this background, it is surprising how little we know about the composition of the microbial communities in indoor environments. The information is fragmentary and mostly based on cultivation methods, although it has been claimed that only approximately 1% of the microbes in indoor environments are viable [7].

Most of the literature about bacteria in normal indoor environments is concerned with cultivation of air samples. Indoor dust samples have rarely been investigated by cultivation or methods permitting identification of bacterial species. In healthy offices, *Enterococcus*, *Staphylococcus*, *Pantoea *and *Pseudomonas *were the most abundant bacterial genera found in carpet dust [8]. Based on the results of cultivation methods, it seems that both Gram-positive and Gram-negative bacteria are common in indoor air of residential settings, office buildings and hospitals [8-11]. There are several frequently encountered taxa; Gram-positive cocci, corynebacteria, bacilli, in addition to Gram-negative species, such as *Acinetobacter *and *Pseudomonas*.

However, Gram-positive bacteria dominate the flora, at least in studies using culture methods, comprising up to 75% of the bacteria present in the indoor air [9]. Certain Gram-positive bacteria with strong immunogenic properties or potential toxin producers, such as mycobacteria, streptomycetes or *Nocardiopsis *spp., have also been shown to be present in indoor environments [12-14]. In a mouse model, these bacteria induced inflammation *in vitro *and *in vivo *[15,16].

Cultivation methods can only detect a small fraction of microbial flora, depending on the culture conditions being used and the viability of the microbes. With respect to indoor environments, the cultivability has been reported to vary between 0.03% to 100%, depending on the sampling method and microbial species, for example [7,17]. Bacteria are less resistant to sampling stress than fungi. It would be predicted that application of molecular methods will change the current picture of what bacteria are present in indoor environments. Sequencing of 16S ribosomal DNA clone libraries has been successfully used for characterisation of bacterial flora in soil, marine habitats and human intestine [18-20]. The advantage of this approach is that all microbial species should be detected equally and the limitations of culture conditions can be overcome. One major benefit is that one can make a relatively exact phylogenetic placement of the detected DNA sequences with respect to reference sequences in public databases. Recent advances in statistical analysis methods of clone libraries [21-23] allow one to conduct a more sophisticated assessment of the differences in microbial communities between samples.

Recently, two studies using culture-independent methods to investigate bacterial flora in indoor environments have been published [24,25]. Lee et al. (2007) investigated the bacterial diversity in a child-care facility using both culture-based and molecular methods. In that study, *Pseudomonadaceae *and *Oxalobacteraceae *were the most dominant families in the clone libraries, though Gram-positive genera, such as *Bacillus*, *Streptococcus *and *Staphylococcus *were also present. The most commonly cultured bacteria were *Bacillus *spp. and *Staphylococcus *spp. The authors found 29 putative bacterial species among the isolates, compared to 141 distinct OTUs using 3% sequence divergence in the clone libraries. Kelley et al. (2004) studied biofilms present on shower curtains using 16S rDNA clone libraries and found that this microenvironment maintains a diverse microbial community. In that study, sequences representing *Sphingomonas *spp. and *Methylobacterium *spp. were the most often encountered. Already these two examples show that the microbial community in indoor environments is more diverse than would have been suspected from reports of culture studies. In a study of the outdoor air microbes present in two cities in USA using molecular methods, the most common bacteria found in the outdoor air were acidobacteria, verrucomicrobia, bacilli, clostridia and some proteobacteria, such as *Sphingomonas*-species [26].

Indoor microbial samples can be taken from surfaces as swab samples, from indoor air or dust. House dust is a reservoir for all kinds of pollutants and can be considered as a long-term integrated sample of indoor particulate matter that has, at least partly, been airborne [27]. Dust is relatively easy to collect for analysis and in the last years, dust samples have been extensively used in epidemiological studies to characterise human exposure to environmental microbial material [28,29], hence dust samples are widely used and provide a good picture of the total microbial exposure in indoor environments. In many epidemiological studies, the endotoxin level in dust samples has been used as surrogate for exposure to environmental microbes. However, measurement of endotoxin activity does not provide an accurate assessment of the total microbial, or even the bacterial exposure. Therefore, more information about the actual species diversity in indoor dust is needed to allow a better assessment of the importance of indoor bacteria for human health. It has been shown that fungal concentrations in indoor air and dust vary temporally [30-32] and thus, the sampling time and season can affect the results of an indoor microbial analysis. Seasonal variation of cultivable levels of bacteria in indoor air has also been observed; though no clear pattern has emerged [9]. Seasonal changes in the bacterial flora of house dust have not been investigated earlier; this is a gap in the fundamental knowledge needed for exposure assessment.

The aim of this study was to investigate the species level diversity and seasonal dynamics of bacterial flora in indoor dust by sequencing of 16S rDNA clone libraries. Dust samples were collected from hard indoor surfaces including floor, tables and shelves by vacuum cleaning. Office rooms in two buildings were sampled at different times during one year to obtain four samples per building, one for each season. Eight 16S rDNA clone libraries were constructed and approximately 100 sequences per library analysed. The results indicated a high amount of Gram-positive species and human-derived sequences in indoor dust and furthermore that variation between buildings was more pronounced that variation between seasons. To our knowledge, this is the first study where full length 16S clone libraries have been used to describe in detail the diversity of indoor bacterial populations.

## Results

### Bacterial diversity in house dust

**Figure 1:**
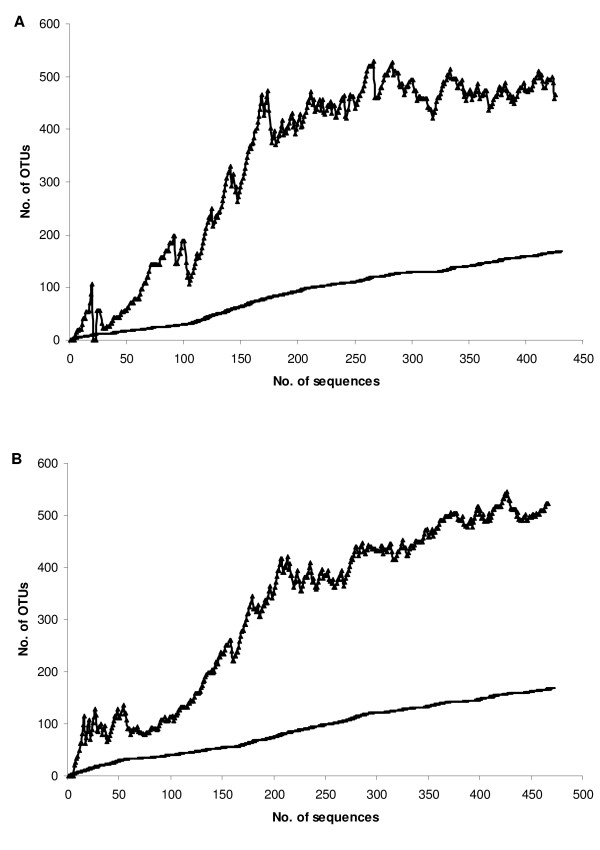
**Collector's curves for the combined sequence datasets for Buildings 1 and 2**. Collector's curves illustrating the observed and estimated OTU richness and the number of sequences sampled in the pooled datasets of Building 1 (A) and Building 2 (B) at the evolutionary distance of 0.03. ▲, estimated number of OTUs; -, observed number of OTUs.

A total number of 953 full-length sequences were obtained from the eight clone libraries derived from the indoor dust of two buildings, one sample representing one season in each building. A total of 60 of these sequences originated from chloroplasts, and were excluded from further analyses. A few chloroplast sequences were observed in all samples; however, they mainly occurred in the summer samples. Altogether 893 clones were further analyzed, among which 283 different OTUs were detected using 3% sequence difference as a criterion. On the average, 112 sequences were obtained and 103 OTUs detected per season. Shannon and Simpson diversity indices indicated the highest species richness in the spring samples, followed by fall, summer and winter samples. In addition, they pointed to a higher diversity in Building 1 during all seasons, except winter (Table 1). ACE and Chao estimators, on the contrary, suggested highest species diversity in the fall sample of Building 1 and in the winter sample of Building 2. The sample coverage for the individual libraries ranged from 19 to 35%. Collector's curves for pooled samples of both buildings are displayed in Figure 1.

**Table 1:** Characteristics of the clone libraries.

**Sample**	**No. of clones**	**S obs**	**S ACE**	**S Chao**	**Coverage ACE (%)**	**Coverage Chao (%)**	**Shannon**	**Simpson**
1W	102	30	147	99	20	30	1.88	0.383
2W	159	53	282	463	19	11	3.18	0.070
								
1Sp	76	59	228	184	26	32	3.96	0.009
2Sp	109	65	188	131	35	50	3.85	0.027
								
1Su	144	60	176	168	34	36	3.47	0.053
2Su	82	45	223	211	20	21	3.28	0.061
								
1F	104	66	305	231	22	29	3.88	0.023
2F	117	55	171	191	32	29	3.27	0.096
								
1 all clones	426	167	465	440	36	38	4.22	0.040
2 all clones	467	167	522	464	32	36	4.10	0.050

The table lists the number of clones, observed and estimated species richness, coverage and diversity indices for the clone libraries from dust samples collected in the two buildings during four seasons. Numbers were calculated with DOTUR program. OTUs were defined using a distance level of 3%. S obs, observed number of OTUs; S ACE, estimated number of OTUs using ACE estimator (Hughes et al, 2001); S Chao, estimated number of OTUs using Chao estimator (Hughes et al, 2001); Shannon and Simpson, diversity indices (Hill et al., 2003) 1, Building 1; 2, Building 2; W, winter; Sp, spring; Su, summer; F, fall.

**Figure 2:**
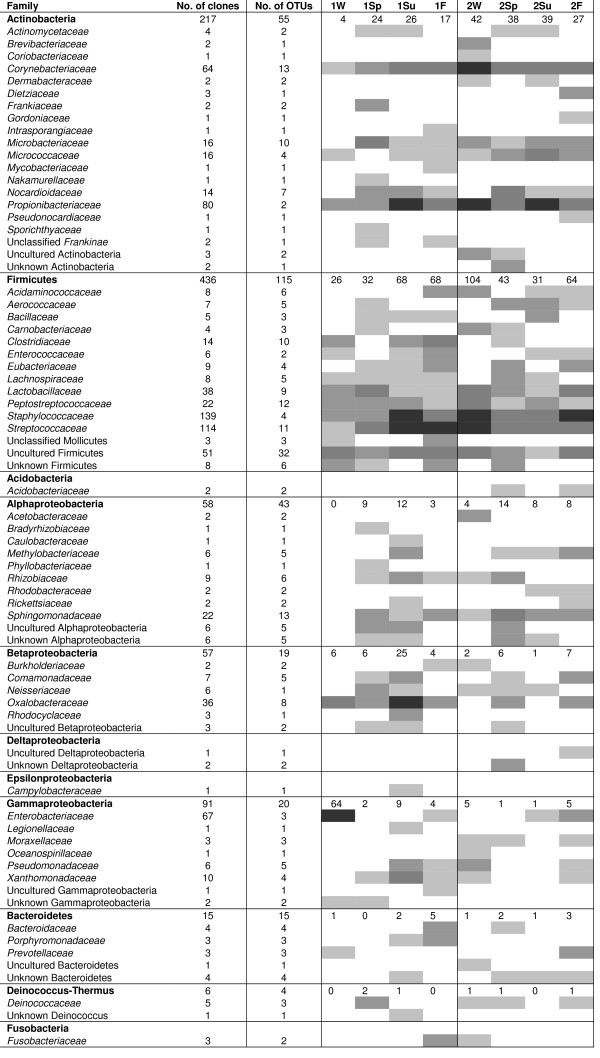
**Abundance of clones and OTUs**. Distribution of the clones and OTUs in taxonomic groups and their abundance in the individual samples are displayed. Number of clones per season in the largest phyla is indicated in the row with the name of the phylum. 1, Building 1; 2, Building 2; W, winter; Sp, spring; Su, summer; F, fall. Uncultured refers to sequences of species not yet cultured; unknown refers to a sequence similarity less than 95% to a database sequence, however if the clone was clearly affiliated to a certain group in the phylogenetic tree, it was assigned to that group. In addition to the clones listed in the figure, there was one clone in Building 2, summer sample affiliating with Planctomycetes, one clone in Building 1, spring sample affiliating with Chloroflexi, and one clone with unknown phylogenetic affiliation in Building 1, winter and fall samples. The grey shading indicates the number of clones in the sample belonging to the respective family. White indicates zero clones, the grey shadings 1, 2–5, or 6–14 clones, and black indicates 15 or more clones.

The bacterial flora in the house dust was dominated by Gram-positive species. In all, 74% of the clones and 60% of the OTUs were assigned to actinobacterial and low-GC Gram-positive species. The most abundant sequences that were also detected throughout the year were those having a high similarity to sequences of *Corynebacterium-, Propionibacterium-, Streptococcus-, Staphylococcus*-, *Lactococcus-, Peptostreptococcus *and *Lactobacillus *species (Figure 2). Many of the low-GC Gram-positive OTUs were similar to sequences from bacteria not yet cultured originating from human samples, such as colon or different mucosa. In addition, sequences close to known genera that are common in human colon, such as *Clostridium*, *Peptostreptococcus *and *Ruminococcus *were abundant in both buildings. These OTUs consisted mostly of single sequences, indicating a high diversity of this kind of phylotypes in the dust. Proteobacterial phylotypes were present in all samples, the most abundant ones being assigned to four families; *Sphingomonadaceae*, *Xanthomonadaceae*, *Oxalobacteraceae *and *Rhizobiaceae *(Figure 2). A few phylotypes affiliating with the phyla *Bacteroidetes*, *Fusobacteria *and *Deinococcus-Thermus *were present in both buildings and single OTUs of the phyla *Chloroflexi *and *Planctomycetes *were found in only one building. Most of the sequences had high similarity to existing sequences in databases; only 8% of the OTUs had a < 95% similarity to any known sequence and about 60% of the OTUs had a sequence similarity > 97% to a known species. Sequences having less than 95% similarity to database sequences were checked for chimeras, but none were found. Detailed information about all the sequenced clones is presented in the Additional file 1.

All of the sequences have been submitted to the European Molecular Biology Laboratory (EMBL) sequence database. The accession numbers are the following; sequences from Building 1, winter sample (clones BF0001A001-102): AM696714–AM696815, Building 2, winter sample (clones BF0002A001-159): AM696816–AM696974, Building 1, spring sample (clones BF0001B001-76): AM696975–AM697050, Building 2, spring sample (clones BF0002B001-109): AM697051–AM697159, Building 1, summer sample (clones BF0001C001-144): AM697160–AM697303, Building 2, summer sample (clones BF0002C001-82): AM697304–AM697385, Building 1, fall sample (clones BF0001D001-104): AM697386–AM697489, Building 2, fall sample (clones BF0002D001-117): AM697490–AM697606.

### Seasonal dynamics

**Figure 3:**
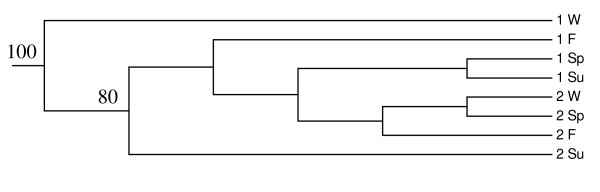
**Cluster analysis of the clone libraries**. The tree was created with Cluster environments analysis of the Unifrac program. Jackknife with 100 permutations was performed to assess the reliability of the tree. Jackknife values over 50 are given at the corresponding branches. 1, Building 1; 2, Building 2; W, winter; Sp, spring; Su, summer; F, fall.

**Figure 4:**
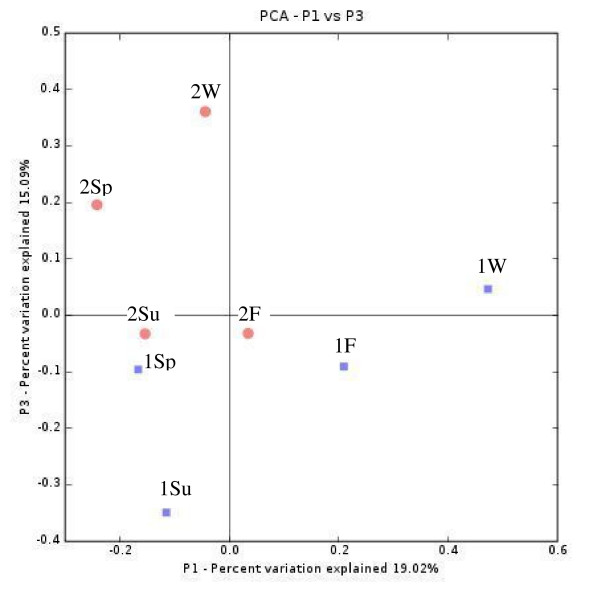
**Principal coordinate analysis**. Unifrac PCA analysis of the eight clone libraries. First principal component (P1) represents the season and P3 represents the building. 1, Building 1; 2, Building 2; W, winter; Sp, spring; Su, summer; F, fall.

The relationships between the clone libraries of individual buildings and seasons were analysed using the Cluster environments-option of the Unifrac program. It seemed that samples from the same building clustered together, rather than the seasons (Figure 3). The samples from Building 2 clustered well together, except for the summer sample. The winter sample from Building 1 differed from all of the other samples from that building, but the remaining samples from Building 2 clustered together. A principal coordinate analysis (PCA) conducted with the Unifrac program, suggested however that 19% of the variation between the samples could be explained by the season and 15% by the building (Figure 4). Neither of the factors separated the samples well; only the winter samples were distinguishable from the others.

Two programs, Libshuff and Unifrac, were used to test the significance of the differences between the samples and the results are displayed in Table 2. In Building 1, only the winter sample differed from the others significantly when Libshuff was used to compare the libraries. When Unifrac was used, the results indicated that all libraries differed statistically significantly from each other, with the exception of the spring and summer samples. In Building 2, the Libshuff suggested that all libraries differed from each other statistically significantly, although only one of the two p-values that the program calculated in each comparison was significant. Using Unifrac, only the winter-summer and winter-fall comparisons were marginally significant (Table 2). The Unifrac significance test results are in line with the Unifrac PCA results (Figure 4). In the PCA plot, the winter and fall samples of Building 1 were well separated along the P1-axis (season), but not the spring and summer samples. The samples from Building 2 are not so well separated from each other along the season-axis.

**Table 2:** Statistical comparison of the clone libraries.

**Samples**	**ΔC_XY _Libshuff**	**p XY Libshuff**	**ΔC_YX _Libshuff**	**p YX Libshuff**	**p Unifrac**
*Comparison of seasons:*					
1W vs. 1Sp	7.302	0.001*	1.269	0.001*	< 0.001*
1W vs. 1Su	5.877	0.001*	1.141	0.001*	< 0.001*
1W vs. 1F	1.649	0.001*	0.841	0.001*	< 0.001*
1Sp vs. 1Su	0.055	0.763	0.103	0.416	0.320
1Sp vs. 1F	0.082	0.446	0.265	0.032	0.01*
1Su vs. 1F	0.066	0.179	0.078	0.150	< 0.001*
					
2W vs. 2Sp	0.106	0.086	0.219	0.002*	0.180
2W vs. 2Su	0.387	0.001*	0.066	0.398	**0.02**
2W vs. 2F	0.033	0.303	0.103	0.024*	**0.02**
2Sp vs. 2Su	0.234	0.013*	0.047	0.576	0.07
2Sp vs. 2F	0.150	0.010*	0.142	0.014*	0.09
2Su vs. 2F	0.034	0.725	0.317	0.002*	0.04
					
*Comparison of buildings:*					
1W vs. 2W	5.989	0.001*	0.407	0.001*	< 0.001*
1Sp vs. 2Sp	0.067	0.598	0.080	0.383	0.16
1Su vs. 2Su	1.177	0.001*	0.050	0.613	0.01*
1F vs. 2F	0.128	0.019*	0.050	0.255	< 0.001*

Comparisons using web-based Libshuff and Unifrac significance analyses were conducted between the individual buildings and the four seasons. ΔC_XY _– and ΔC_YX _– values represent the difference between the two libraries calculated by Libshuff. Libraries that are significantly different with a confidence of 95% are marked with*. 1, Building 1; 2, Building 2; W, winter; Sp, spring; Su, summer; F, fall.

The seasonal fluctuation in the actual bacterial flora within one building at the species level was large; less than one percent of the OTUs were found in more than two seasons and over 75% of all OTUs in one building were found in only one sample. Therefore, differences at the higher taxonomic levels, such as the class level, were investigated. The percentual proportion of low GC Gram-positive and actinobacterial OTUs remained almost the same during all seasons in Building 2. With the exception of a slight decrease in alphaproteobacterial and an increase in the gammaproteobacterial diversity in the winter sample, the differences between seasons were small. In Building 1, the seasonal differences in the proportions of different bacterial classes were clearer. The amount of actinobacterial OTUs was higher in the spring sample and that of alpha- and betaproteobacteria in the summer sample. The numbers of alpha- and betaproteobacterial OTUs were highest in spring and summer samples in both buildings.

### Comparison of the two buildings

When combining all sequences obtained from one building into a composite sample and comparing the two buildings with each other with the Libshuff program, the libraries were statistically different (Table 2). This difference was confirmed by both Libshuff and Unifrac in comparisons of the buildings over the individual seasons, with the exception of spring (Table 2). In the Unifrac cluster environments and PCA analyses, the buildings were clearly separated from each other (Figures 3 and 4). The Shannon and Simpson diversity indices pointed a higher diversity in Building 1 throughout the year, except in winter.

From the 283 OTUs found, 56 were present in both buildings; 15 of them were of actinobacterial origin, 28 firmicutes, six were alphaproteobacteria, five OTUs were affiliated with betaproteobacteria and two with gammaproteobacteria. The actinobacterial OTUs comprised sequences with similarity to coryne- (98–99%), propioni- (99%), and microbacteria (96–97%) as well as micrococci (96–99%), *Actinomyces viscosus *(98%), *Nocardioides oleivorans *(99%) and *Friedmanniella spumicola *(99%). The OTUs affiliating with the phylum *Firmicutes *included mostly OTUs having their closest matches among staphylo- (99%), strepto- (99%) and peptostreptococci (96–99%), lactobacilli (99%) and uncultured species from human colon (97–99%), in addition to some OTUs having 99% sequence similarity to *Ruminococcus*, *Enterobacterium*, *Enterococcus*, *Selenomonas *and *Veillonella *species. The alphaproteobacterial sequences present in both buildings were similar to *Methylobacterium organophilum *(97%), *Rhizobium *(97–98%) and *Sphingomonas *species (98–99%), betaproteobacterial sequences affiliated with *Neisseria *spp. (99%), *Variovorax *spp. (99%) and *Janthinobacterium *spp. (97%) and gammaproteobacterial OTUs had a high sequence similarity to *Shigella sonnei *(99%) and *Stenotrophomonas maltophilia *(99%). Four OTUs were observed throughout the year in both buildings. These had 99% sequence similarity to *Corynebacterium *spp., *Propionibacterium acnes*, *Staphylococcus epidermidis *and *Streptococcus thermophilus *(see Additional file 1).

Differences in the bacterial flora between the two buildings were mainly due to OTUs consisting of one sequence; in all samples, there were 14–27 singletons that explained 66–74% of the variation between buildings. If it is assumed that occurrence of one sequence type in several libraries from the same building indicates that it originates from an indoor source, there were few such OTUs present, and none of them were present in all four samples. With respect to the sequence types that were present during more than one season, 14 were detected only in Building 1. These were clones affiliating with Microbacteriaceae, unclassified Frankinae, Rhizobiaceae, Oxalobacteraceae, Clostridiaceae, Staphylococcaceae and uncultured Firmicutes. The only phylotype in Building 1 detected in three samples had 99% sequence similarity to *Bacillus cereus*. In Building 2, there were 13 OTUs detected only in that building in more than one sample; for four of them their closest matches were *Kocuria palustris *(99%), *Aerococcus viridans *(99%), uncultured *Deinococcus *(96%) and uncultured bacterium from human vagina (98%), were detected in three samples.

## Discussion

The molecular diversity of the bacterial flora in indoor environment was monitored in two buildings over all four seasons using 16S rDNA clone libraries derived from dust samples. As far as we are aware, this is the first study to have used 16S clone libraries in the characterization of the indoor dust bacterial flora. Two other papers have been recently published, one investigating shower curtains and one studying cotton swab surface samples from a child-care facility [24,25]. In this work, dust was chosen as the sample material because it is widely used in studies involving indoor environments and can be considered as a long-term integrated sample of the pollutants that have been airborne [27]. The disadvantage of dust samples is that they are a complex mixture of microbes originating from many different sources including the outdoor air. However, this study intended to gather basic information about the bacterial flora present in an indoor environment with all the possible sources, and dust samples serve that purpose well. Dust was collected only from hard surfaces, such as hard floor, tables and shelves, since carpets and textile covered furniture can retain microbes and interfere with the assessment of seasonal variation. Altogether 893 clone sequences were analyzed and within them, 283 distinct OTUs were detected using 3% sequence difference as the criterion. Lee *et al*. (2007) sequenced 453 clones in cotton swab samples from a child-care facility and found 141 bacterial species using the 3% divergence level, i.e. almost exactly the same proportion as in our study. The Shannon and Simpson diversity indices suggested highest diversity in the spring samples and lowest in the winter samples in both buildings. The Shannon index gives more weight to the rare species and Simpson to the dominant [33], but in this case they were concordant. The ACE and Chao estimators did not agree with Shannon and Simpson in all cases. The Chao estimator takes into account only singletons and doubletons, ACE uses OTUs having one to ten clones each [34]. The ACE and especially Chao are dependent of the amount of singletons and the discrepancies with the diversity indices are most probably due to different amounts of singletons in the libraries. The estimated coverage of the libraries varied between 19 and 40% when using the ACE estimator. The coverage values are higher than those reported for clone libraries from soil, where library coverages from 7 to 16% have been described [18,35]. In addition, higher coverages have been reported with libraries from human sources, *e.g*. 76–86% [36] and as high as 99% [20], which may be due to the larger number of sequenced clones in the latter studies.

In both buildings investigated in this work, the estimated OTU number was about 500 using 97% sequence identity as the criterion in DOTUR (Distance-based OTU and richness), and the pooled sequence data from all four samples taken from that building. The ACE estimate for the individual libraries varied from 147 to 305. The individual libraries harboured many sequence types unique to that library, so the pooled data set including all seasons provides a better estimate of the total diversity in that building than a single sample. In clone libraries, with increasing numbers of sequences, the number of OTUs increases, until saturation is reached. For the pooled datasets, the saturation in the estimated total OTU number was more or less achieved, for Building 1 better than for Building 2, as shown in the collector's curves. Thus, 500 OTUs represent a relatively good estimate of the number of bacterial species in house dust.

### Bacterial diversity in house dust

In our study, Gram-positive bacteria, especially bacteria of the phylum *Firmicutes *dominated the flora. This is in accordance with culture-based studies made in indoor environments. The most frequently encountered sequences in this work originated from species of the genera *Corynebacterium*, *Propionibacterium*, *Staphylococcus *and *Streptococcus*. In a study of indoor air of residential buildings, staphylococci accounted for 16–37% of the culturable bacteria, depending on the room where samples had been taken [9], i.e. the proportion was highest in the bedroom and lowest in the basement. Bouillard *et al*. (2005) investigated healthy office buildings, and found that the two most frequently found species in air samples were *Micrococcus *spp. and *Staphylococcus *spp. and in addition, bacteria of the family *Streptococcaceae *were present in dust samples. These abundant bacteria most probably originate from the users of the building as these species are typical representatives of the normal flora of human skin, outer ear and oral cavity [37-40]. Moreover, there was a multitude of phylotypes affiliating with species or phylotypes abundant in human colon or feces, such as *Bacteroidetes*, *Clostridium*, members of the family *Peptostreptococcaceae *and a number of species not yet cultured. This emphasizes the major human impact on the indoor dust microbiota, at least in these two buildings. The high diversity of bacteria from human sources may also reflect the high occupancy of the buildings by many different persons. Although the sampled rooms were only used by the personnel, carryover from the rooms used by patients may also have occurred. A molecular survey of aeroplane bacterial contamination revealed that the same Gram-positive genera; *Streptococcus, Staphylococcus, Corynebacterium Propionibacterium *and *Kocuria *that are common in indoor dust are present in the indoor air and surfaces of aeroplanes [41].

Approximately 40% of the OTUs were of Gram-negative origin. The most abundant families in this study were *Sphingomonadaceae*, *Xanthomonadaceae*, *Oxalobacteraceae *and *Rhizobiaceae*. These bacteria are common in the soil and rhizosphere, however, some species, such as *S. maltophilia*, are potential pathogens. In the study of Bouillard *et al*. (2005), Gram-negative species accounted for approximately one-third of the total bacterial strains isolated from air, dust and surface samples of an office building, with the two dominant species being *Pantoea *sp., *S. maltophilia *and *Pseudomonas putida*. On the contrary, the culture-independent studies have suggested that Gram-negative species are more abundant than the Gram-positive species. Lee *et al*. (2007) found that *Pseudomonas*- and *Oxalobacteraceae*-like sequences were the most abundant in their clone libraries from a child-care facility. This may be due to the different DNA extraction method used; in our study, we used bead-beating, whereas Lee *et al*. used enzymatic cell lysis, which is a more gentle method and may favour extraction of the DNA from gram negative bacteria. The bead beating time used in the DNA extraction protocol may also affect the observed community composition [42]. Gram-negative bacteria of the orders *Burkholderiales *and *Sphingomonadales *were more readily detected with a microarray after 5s bead beating. In contrast, Gram-positive bacteria were better detected after 45s bead beating [42]. The differences can also be attributable to the different sample types; the swab samples taken from toy and furniture surfaces in the study of Lee *et al*. (2007) probably harboured mostly bacteria able to form biofilms on these surfaces. In addition, Kelley *at al*. (2004) reported that *Sphingomonas *spp. and *Methylobacterium *spp. were frequently present in biofilms on shower curtains. These genera were also found in this study, in both buildings, which suggests that they are members of the normal microflora of buildings.

### Seasonal dynamics

Information about the seasonal variation is important for any exposure assessment. To date, most of the published information about seasonal variation of microbial flora in indoor environments has concentrated on viable counts of fungi, and to a lesser extent, on bacteria. Viable fungal concentrations in house dust are known to vary between seasons [32], however the seasonal variation of the viable flora at the species level is not very clear [31]. The first study using molecular methods to investigate the seasonal variation of fungal flora in indoor environments has been recently conducted by Pitkäranta et al. [43]. Seasonal variation of indoor bacterial concentrations has been studied using air samples. The study by Reponen *et al*. (1992) showed that although the indoor air counts of fungi were significantly lower in wintertime than during other seasons, airborne bacteria did not exhibit an equally clear seasonal pattern [44]. Moschandreas *et al*. (2003) found that total concentrations of cultivable bacteria in indoor air of 20 homes in the Chicago area were highest in summer and fall [9].

We investigated the seasonal variation of the bacterial flora using statistical tools. The two programs used for the analyses, web-Libshuff and Unifrac-significance, gave rather similar outcomes and only in a few cases contradictory results. In Building 1, only the winter sample differed from the other samples when using the Libshuff analysis. Unifrac detected a statistically significant difference between all seasons, except for spring and summer. In Building 2, both programs detected differences between some seasons, but they were not highly significant. These findings are supported by the Unifrac cluster environments and PCA analyses, in which the seasons were not clearly distinguished apart from the Building 1 winter sample Building 2 summer sample. The clear distinction of the Building 1 winter sample is mainly due to dominance of one OTU type in the library having 99% sequence similarity to *Serratia fonticola*, a member of the enterobacterial group, and so represented a building-driven trend, rather than seasonal. The dominance of this OTU may reflect a temporary source present at the time of the sampling, or it may be a bias inherent in polymerase chain reaction (PCR)-technology. It is known that some sequences may be preferentially amplified in PCR resulting in a higher dominance of these sequences in the library [45]. To prevent this phenomenon, all amplifications were done in 10 replicates and the number of cycles was kept low. Since *S. fonticola *was observed only in one sample, we assume that it represented a temporary source. In the PCA analysis, 19% of the variation between samples was explained by the season and 15% by the building. However, the seasonal difference is driven by the winter sample of Building 1, which has the above mentioned problems and otherwise the differences were not very large. The comparisons between buildings were all statistically significant and the seasons of the same buildings also clustered together, thus it does seem that the differences between buildings are more consistent. Although the seasonal variation could not be clearly demonstrated with the statistical methods used, a strong seasonal variation existed at the species level, since more than 75% of the OTUs detected in one building were present in only one season. The statistical methods are based on evolutionary distances; Libshuff uses a distance matrix and Unifrac a phylogenetic tree as the input, and probably reflect better changes in the community structure than differences on species level.

Comparison of the samples at a higher taxonomic level to some extent revealed an increase in the relative abundance of alpha- and betaproteobacteria towards summer at the expense of low GC Gram-positive bacteria. The proportion of low GC Gram-positive bacteria and gammaproteobacteria in the total diversity was highest in winter, that of actinobacteria, alpha- and betaproteobacteria in spring or summer, and finally the diversity of bacteroidetes peaked in the autumn. This may reflect the diverse sources of these bacteria and their fluctuating impact on the indoor microbial flora. The species of the phyla *Firmicutes, Gammaproteobacteria *and *Bacteroidetes *detected in this study contained mostly those that are normal inhabitants of the human body [1]. Since the proportion of low GC Gram-positive bacteria and gammaproteobacteria was highest in winter, it seems that the human impact on the microbial flora is highest in winter. Alternatively, the human impact is constant, but during other seasons outdoor factors have a greater influence on the microbiota. Some of the actinobacterial phylotypes encountered, such as *P. acnes *and some corynebacteria, are most probably of human origin, but other types detected in spring and summer samples, such as OTUs similar to the genera *Frigoribacterium*, *Subtercola *and *Plantibacter *may have originated from outdoor sources. This, as well as the increasing number of Alpha- and Betaproteobacterial sequences in spring and summer perhaps reflected the higher impact of outdoor sources in spring and summer. The results are concordant with the seasonal variation of fungal flora in the same samples [43]. In subarctic climate, the ground is covered by snow in winter and microbial concentrations in the outdoor air are lower than during other seasons. Because of the cold, the windows are also not opened in winter as much as during other seasons. So, the outdoor air affects less the indoor environments in winter, and the indoor sources (human, etc.) can be better detected.

### Comparison of the buildings

The two buildings chosen for the study were similar in age, building frame, ventilation type, use and rural location. The main difference between the buildings was their different status with respect to moisture damage and perceived indoor air quality. A statistical comparison of the two buildings suggested that there were significant differences in the microbial flora of the buildings, apart from the spring samples. The differences in the microbiota of the buildings were mainly driven by singletons. Against that background we cannot say if the observed differences are a reflection of a too small number of clones sequenced, or real differences. Both buildings had bacterial taxa that were typical for that building, and which were detected during more than one season. Some of these are likely linked with the inhabitants of the building (*Staphylococcus*, *Clostridium, Aerococcus*) and some are known to associate with plants (Frankinae, Rhizobiaceae) and thus, may be from outdoor sources or indoor plants. *B. cereus *and the family *Oxalobacteraceae *present in Building 1 were the only OTUs that could be associated with the moisture damaged building. To date, the sole bacterial group that has been associated with moisture damage is the group of spore-forming actinomycetes [46,47]. Spore-forming actinomycetes were found in this study as singletons in some of the libraries; and thus it was not possible to draw a direct connection to the building. *B. cereus *has been isolated from indoor dust of schools and day-care centres [48]. Bacilli and oxalotrophic bacteria are known to colonise fungal hyphae in soil [49]. In theory, these bacteria could be evidence of fungal growth in the building, but for the present, this remains only a hypothesis. In general, because only two buildings were investigated in this study, no conclusions can be drawn regarding the effect of moisture damage on the bacterial flora in this material.

## Conclusion

In conclusion, this work demonstrated that the microbial flora of indoor dust was complex and dominated by Gram-positive species. The dominant phylotypes most probably originated from users of the building. A seasonal variation was observed, this being reflected in the proportional changes of the microbial phyla as well as at the species level. Statistical methods did not detect clear differences between seasons. The microflora of the two investigated buildings differed statistically and differences between the buildings were more pronounced than the differences noted between seasons.

This work provided basic information about bacterial diversity in indoor dust and its seasonal dynamics and hence, qualitative information about the total bacterial exposure in indoor environments. Future work should include a characterisation of the different sources of microbes in indoor dust and a quantitative assessment of the different microbial taxa.

## Methods

### Dust sampling

Dust samples were taken from office rooms of two buildings located in small towns in central Finland about 100 km apart. The buildings were chosen based on their similar age, structure, usage and willingness to participate in the study. A technical inspection was performed in both buildings by a trained civil engineer. Both buildings were brick-framed, had two floors in addition to a basement floor, which was partly underground, and a mechanical exhaust ventilation system. Both were used as nursing homes for the elderly. The sampling was done during the year 2003, and the temperature varied in central Finland as follows: in winter (January-March) the average monthly temperature was -15°C to -2°C, in spring (April-May) +1°C to +10°C, in summer (June-August) +13°C to +21°C and in fall (September-November) +1°C to +11°C. The values are average monthly temperatures measured at Kuopio airport. The Buildings 1 and 2 were located within 60 km radius from the weather station at the airport.

Building 1 was built around 1920, and no substantial repairs had been done since that time. The basement floor had undergone some repairs after detection of moisture and microbial damage on the floor and outer walls in 1999. There were also local signs of moisture and microbial damage in the bathrooms in first and second floor. The first floor of the building served as a nursing home and the second floor as a ward of the health centre, housing mostly elderly patients. The employees in the building complained of building-related symptoms and indoor air problems.

Building 2 was a nursing home built around 1940 and which had undergone a thorough restoration 1982. No visible signs of moisture or microbial damage were detected, apart from minor signs in the washroom. The users of the building did not report any problems related to the building or indoor air.

The sampling was performed in the office rooms located on the second floor in both buildings. In both buildings, the sampled rooms had workplaces for two or three persons; however, several other individuals visited the rooms every day. For one sample, settled dust was collected for 2 months, twice a week, from the hard floor and other hard surfaces, such as bookshelves and the tops of cupboards, with a vacuum cleaner Miele S371, (Miele & Cie. KG, Gütersloh, Germany). Separate vacuum cleaners were used for each building, and the tubing and nozzle were cleaned between samples with 70% ethanol. Four samples were collected from each building, corresponding to the four seasons. Hair and larger particles were removed by passing the dust through an autoclaved tea sieve. The remaining fine dust was divided into aliquots and stored at -20°C.

### DNA isolation

Duplicate DNA isolations were conducted from 25 mg of dust using GenElute™ Plant Genomic DNA Miniprep Kit (Sigma-Aldrich Chemie Gmbh, Steinheim, Germany). The sample was weighed into a 2 ml screw cap tube with 0.5 g of 0.1 mm glass beads (BioSpec Products Inc., Bartlesville, OK, USA), 400 μl of lysis solution was added, and the cells were disrupted with Mini Beadbeater-8 (BioSpec Products Inc., Bartlesville, OK, USA) for 1 min at maximum speed. The released DNA was purified with the kit according to the manufacturer's instructions. An additional purification step was performed with a Wizard DNA Clean up column (Promega, Madison, WI, USA) and the DNA was eluted in 50 μl of nuclease free water. The replicates were combined to give 100 μl of DNA originating from 50 mg of dust.

### Construction of clone libraries and sequencing

Universal PCR amplification of the full length bacterial 16S rRNA gene was carried out using primers pA and pH', which target the regions 8–24 and 1522–1542 of the *E. coli *16S rRNA gene, respectively [50]. Ten parallel reactions and negative controls containing nuclease free water instead of DNA in a volume of 50 μl were carried out under the following conditions: 1 × Biotools buffer (B&M Labs, Madrid, Spain), 200 μM each dNTP, 0.2 μM each primer, 5% Dimethyl sulfoxide (DMSO), 0.5 mM Betaine, 2.5 U Biotools DNA polymerase (B&M Labs, Madrid, Spain), 0.05 U Pfu polymerase (Promega, Madison, WI, USA) and 1 μl DNA template. PCR amplification was carried out under the following cycling parameters: initial denaturation at 95°C for 3 min, followed by 25 cycles of 30 s at 96°C, 45 s at 55°C, and 2 min 30 s at 72°C, followed by a final extension at 72°C for 10 min. PCR products were purified with Wizard PCR preps (Promega, Madison, WI, USA) and checked in agarose gel electrophoresis.

Immediately before ligation the purified PCR products were incubated in 72°C for 30 min in the following reaction mix: F-516 DyNAzyme PCR Buffer (Finnzymes, Espoo, Finland), dNTPs and DyNAzyme DNA Polymerase (Finnzymes, Espoo, Finland) in concentrations mentioned above. Using PCR Cloning Kit (Qiagen, Hilden, Germany) according to the manufacturer's instructions, 4 μl aliquots of PCR products were ligated and cloned into the competent *E. coli *provided in the kit. Minimum 192 white colonies per library were picked and grown overnight in 1 ml of LB-broth [51] containing 150 μg/ml ampicillin. Aliquots of clone cultures were stored with 15% glycerol at -80°C and plasmids were extracted from the remaining culture using MultiScreen _96_PLASMID Plates (Millipore, Billerica, MA, USA). Inserts were re-amplified with universal forward and reverse primers targeting the vector sequences flanking the insert. PCR products were visualised in 1% agarose gel with ethidium bromide staining and purified with MultiScreen _384_PCR-plates (Millipore, Billerica, MA, USA).

Purified fragments were sequenced using the BigDye Terminator cycle sequencing kit version 3.1 (Applied Biosystems, Foster City, CA, USA) with internal primers pD', pE and pF'. Sequencing reactions were run on ABI3700 automated DNA sequencer (Applied Biosystems, Foster City, CA, USA).

### Sequence analysis

Sequences were edited and assembled using Pregap and Gap4 programs of the Staden Package [52]. Full length sequences excluding primer sites were aligned against EMBL DNA databases using Fasta 3.4 [53]. Sequences having less than 95% similarity to known sequences were checked for chimeras using the chimera detection available at the Ribosomal Database project website [54]. Multiple alignments of the sequences were constructed using AlignX program of the VectorNTI package (InforMax Inc., Bethesda, USA). ClustalW and DNADIST program available in the PHYLIP package [55] were utilised to construct a distance matrix that was used as an input in the DOTUR-program, which was used for defining OTUs at a distance level of 3%.

Sample coverages and species richness estimates were calculated using the DOTUR program [23]. The program calculates various diversity indices and species richness estimators at different distance levels. The estimates were calculated separately for each season and building sample as well as for a pooled sample containing the libraries from both buildings collected during one season and thus, representing that season.

The sequence libraries obtained from each building and season were compared using web-Libshuff version 0.96 and Unifrac. Web-Libshuff is a web-based version of the Libshuff program [21]. The program compares two sequence libraries to determine if they differ significantly from each other. The program calculates a homologous and a heterologous coverage curve for the libraries, uses Cramér von Mises statistic to calculate the distance between the two curves and Monte Carlo test procedure to compare them. Unifrac can compare several libraries at the same time and can also be used to visualise relationships between environments [22]. The two buildings were compared for each season and the seasons were compared within each building.

## Authors' contributions

HR performed the data analysis and drafted the manuscript, MP did the cloning and sequencing and edited the manuscript, MT collected the samples and inspected the buildings, LP supervised the sequencing and edited the manuscript, AN designed the study and edited the manuscript. All authors read and approved the final manuscript.

## Supplementary Material

Additional file 1 **Assignment of the individual clone sequences to OTUs, phylogenetic affiliation and occurrence of the OTUs**. Table displays all OTUs found in this study and all clones assigned to the respective OTUs based on the DOTUR program and 97% sequence similarity. The closest database match and the phylogenetic assignment are based on the sequence of the first clone listed in the respective OTU and listed in the column as the Representative clone. The occurrence in the two buildings and in different seasons is also indicated. 1, Building 1; 2, Building 2; W, winter; Sp, spring; Su, summer; F, fall; 1, occurs only in Building 1; 2, occurs only in Building 2; B, occurs in both buildings. Click here for file
